# Scratching the Surface of Psychiatric Services Distribution and Public Health: an Indiana Assessment

**DOI:** 10.1007/s11414-018-9626-7

**Published:** 2018-06-26

**Authors:** Steven Moberly, Hannah Maxey, Lacy Foy, Sierra X. Vaughn, Yumin Wang, David Diaz

**Affiliations:** 1 Department of Psychiatry, Indiana University School of Medicine, Indianapolis, IN USA; 2 Department of Family Medicine, Indiana University School of Medicine, Indianapolis, IN USA; 3 Bowen Center for Health Workforce Research and Policy, 1110 W. Michigan Street, LO 200, Indianapolis, IN 46202 USA; 4 Department of Biostatistics, Indiana University Richard M. Fairbanks School of Public Health, 410 W. TenthSt., Suite 3000, Indianapolis, IN 46202 USA

## Abstract

Mental illness is a leading cause of disability with many public health implications. Previous studies have demonstrated a national shortage of psychiatrists, particularly in rural areas. An analysis of how this workforce distribution relates to population demographics and public/behavioral health is lacking in the literature. This study encompassed a statewide assessment of the Indiana psychiatric workforce as it relates to population characteristics and public/behavioral health. This study’s findings demonstrate a profoundly low psychiatry workforce in rural counties of Indiana. The low psychiatry workforce capacity in rural counties is so disparate that the demographic and public/behavioral health characteristics differ from the State averages in the same manner as counties without a psychiatrist at all. The psychiatric workforce distribution did not differ significantly on the basis of poverty prevalence. The potential utility of indicators of population health was also evaluated and revealed that social factors such as poverty and Medicaid prevalence may be superior to more traditional measures.

**Electronic supplementary material:**

The online version of this article (10.1007/s11414-018-9626-7) contains supplementary material, which is available to authorized users.

## Introduction

Evaluating the distribution of psychiatrists and the services they perform is important to understanding the structure of a mental health system. The World Health Organization recognizes mental illness as the leading cause of disability in developed countries, and previous studies have estimated the economic impact of mental illness in the United States at $300 billion in 2003.^1–3^

In addition to the direct suffering of those afflicted, there are many indirect ways that mental illness affects societies. Mental illness exacerbates morbidity from other chronic diseases (e.g. diabetes, cardiovascular disease, and asthma).^4–7^ Depression alone has been associated with an approximately two- to threefold increase in overall healthcare costs among populations with similar levels of medical comorbidity.^8,9^ Increased rates of homelessness and incarceration as well as more lengthy and expensive hospital admissions are observed among those with mental illness.^8,10–13^ Furthermore, the prevalence of addiction is considerably higher and the complications of addiction are many among those with mental illness.^14–16^ Psychiatrists have a multifaceted role in the provision of mental health services, including differentiating primary mental illness from other medical disease, diagnosis, and overseeing treatment for those with mental illness. In order to better address the burden of mental illness, it is necessary to understand the current structure of psychiatric services as they relate to population health.

Several studies have demonstrated a shortage of psychiatrists and other mental health professionals at the federal, state, and county level. There have been particular shortcomings in recruiting and maintaining psychiatrists in rural areas.^17–21^ While some regions of Indiana have the federal designation of Mental Health Professional Shortage Area, detailed analysis regarding the distribution of psychiatrists and the services they provide is lacking in the peer reviewed literature. Further characterization of the psychiatric workforce as it relates to populations is imperative for guiding the implementation of informed and sustainable changes.

This study was designed to better characterize the structure of the psychiatry workforce and develop a basis for evaluating the interface of this workforce with diverse populations of Indiana. There is specific focus on rurality and poverty as these population characteristics may affect access to medical care. Data were collected at the time of medical licensure to identify primary practice characteristics of physicians licensed and practicing psychiatry in Indiana. These data were analyzed and incorporated with census and public health information to investigate the interface of psychiatry and population health. Furthermore, it was hypothesized that suicide incidence and infant mortality rate would be key indicators of more general public/behavioral health in Indiana. This analysis also leads to further evaluation of social factors as indicators of public and behavioral health outcomes.

## Methods

### Data sources

Indiana has made efforts over the last several years to improve the availability and accuracy of workforce supply data collected from licensed health professionals. The Indiana Professional Licensing Agency has collaborated with [BLINDED] to establish standard survey instruments which are administered to health professionals during biennial license renewal cycles. These instruments collect information regarding demographics, education, and practice characteristics. Survey data are coded and stored in a longitudinal database designed to support health workforce analyses.

**Fig. 1:**
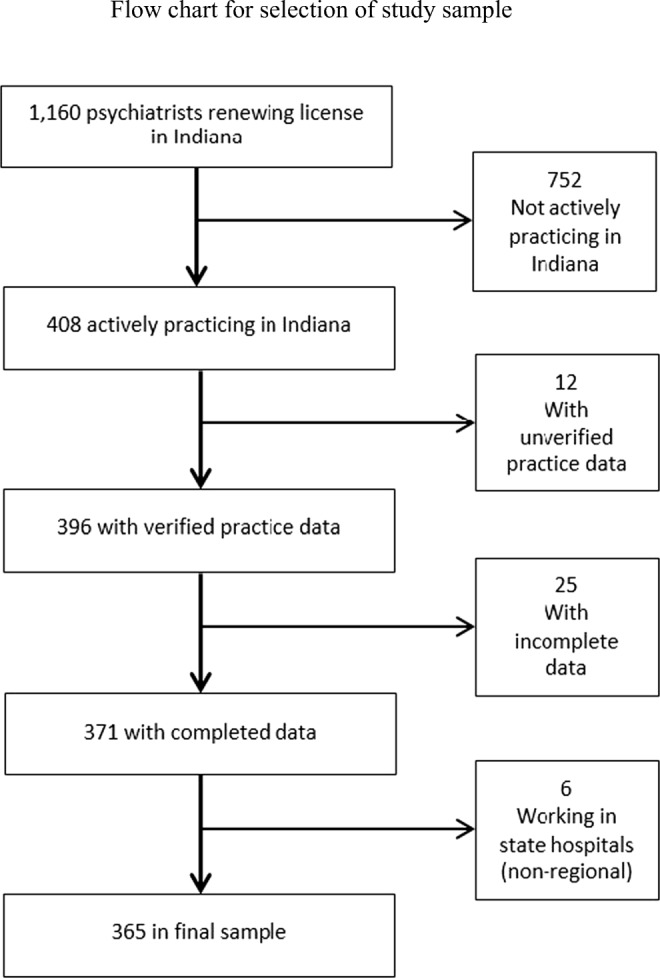
Flow chart for selection of study sample

Psychiatry workforce supply data were collected from the 2015 physician license renewal survey. Additional data collection was conducted to verify practice characteristics for all psychiatrists actively practicing in Indiana. All psychiatrists whose primary practice address could be associated with a single county were considered regional providers and were included in this study. Psychiatrists whose primary practice data could not be verified and those without complete primary practice data were excluded from the study. Also excluded from the study were psychiatrists practicing primarily at a state hospital. The geographic service area for these psychiatrists could not be determined as state hospitals serve patients throughout the state. Of the 1160 psychiatrists who renewed their license in 2015, 365 were actively practicing in Indiana and included in study analyses. Psychiatrist level data on psychiatric specialty, full-time equivalency (FTE), and practice address were included in this study. Figure 1 presents the inclusion and exclusion criteria.

Population characteristics were obtained from several sources. Demographic and socioeconomic estimates were obtained from the American Community Survey (U.S. Census). Data regarding alcohol and substance abuse rates, suicide incidence, and infant mortality rates were obtained from the Indiana State Department of Health.^22–24^ Hypertension prevalence data were obtained from the Center for Disease Control and Prevention.^25^ Smoking and diabetes prevalence data were obtained from the Robert Wood Johnson Foundation’s health rankings data.^26^ Detailed definitions for all variables included in the study analysis are presented in Supplement Table 1.

### Data analysis

Spatial analyses were performed to visualize the distribution of psychiatrists in relation to population size and rurality using ArcGIS (Redlands, California USA). For spatial analysis, psychiatrist full-time equivalency data were aggregated to the zip code level, civilian population count data were aggregated to the county level, and rurality was assigned at the county level using a four-level categorical value representing quartiles of population size.

Descriptive statistics, including count, frequencies, percentages, and means (± standard error), were generated to determine trends in the data at the state and county levels. Descriptive statistics were aggregated at the county level to support comparison between counties with and without psychiatry workforce capacity.

Two-way ANOVA was used to compare mean differences in psychiatry workforce capacity and composition by rurality and poverty. For these analyses, data were stratified by poverty quartile and percent rural quartiles. Pearson’s correlation coefficients were calculated at county level. Statistical analysis was completed using SAS 9.4 software (Cary, North Carolina USA).

## Results

**Fig. 2:**
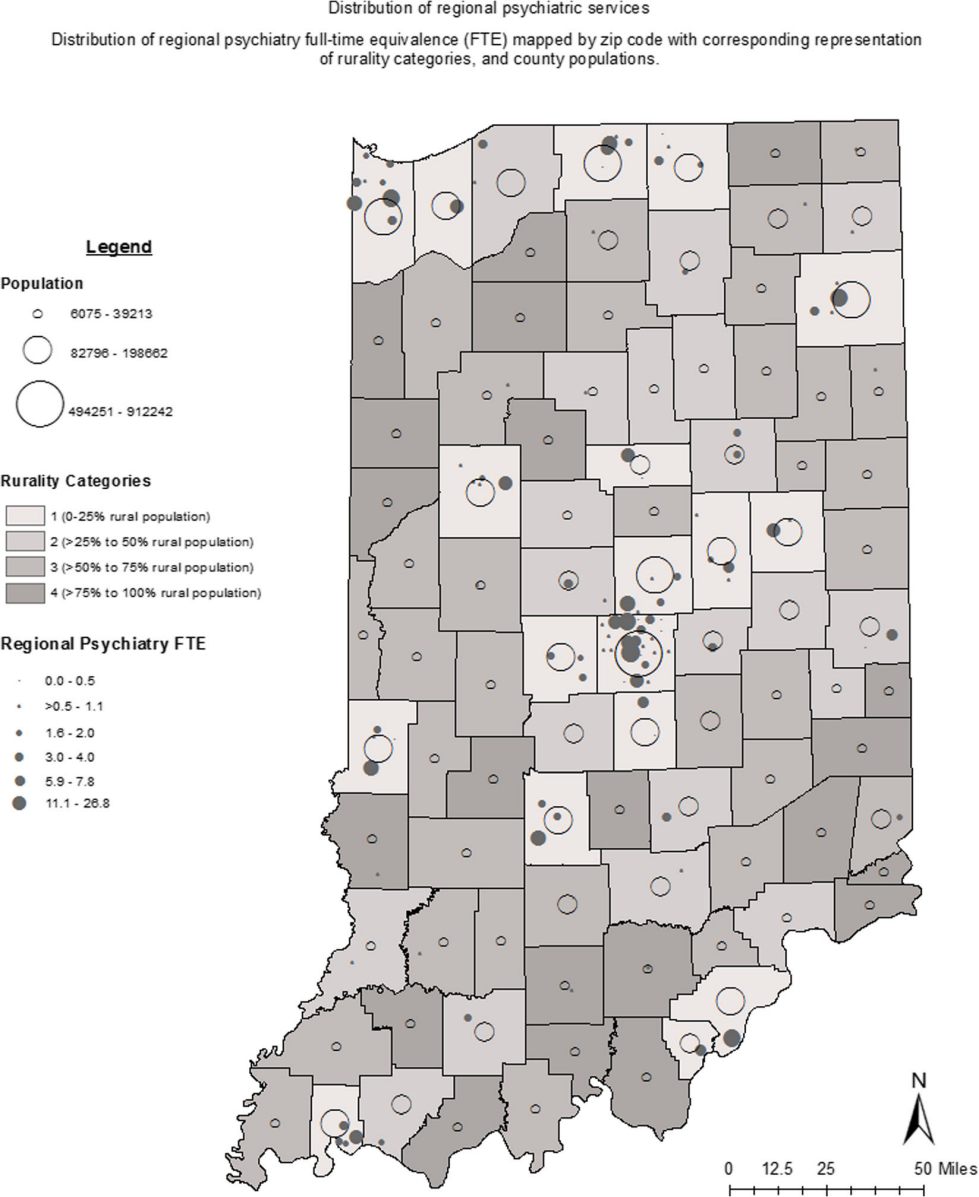
Distribution of regional psychiatric services. Distribution of regional psychiatry full-time equivalence (FTE) mapped by zip code with corresponding representation of rurality categories, and country populations

State level population characteristics as well as county level public/behavioral health measures for Indiana are presented in Table 1. Indiana is a state of over 6.5 million people. While the majority of Indiana’s population resided in urban/metropolitan areas, over 27% (nearly 2.4 million people) were identified as residing in a rural area at the time census data were collected. Thirty-five percent of the state’s population lived in a household that falls at or below 200% of the Federal Poverty Level (FPL), and 21% of the population were Medicaid recipients. The results demonstrated a clustered distribution of psychiatrists in urban/metropolitan areas (Fig.2 and Supplemental Figure 1 A-C) consistent with results from a previous national study.^18^ Less than half of Indiana counties had an actively practicing psychiatrist, and far fewer had psychiatrists who reported a subspecialty practice in geriatric, child-adolescent, or addiction psychiatry (Table 2). Counties without a practicing psychiatrist had a significantly greater percent of the population that lived in a rural area (Table 3). Other factors characterizing the population in these counties more closely compare with the populations characteristics of counties with high rurality (Rural Categories 3 and 4). Such examples of this similarity are higher incidence of suicide, smoking, diabetes, and lower incidence of alcohol abuse (Tables 3 and 4). The percent of the population at or below 200% poverty and the percent of Medicaid recipients were not significantly different between counties with and without reported psychiatric practice (Table 3) and did not correlate with rurality at the state level (Table 4).

**Table 1:** State-wide population characteristics

Total population	6,514,861 individuals
% population residing in rural area	27.4
% population income less than 200% Federal Poverty Line	35.0
% population age 65 or older	13.3
% population under 18 years of age	24.5
% Medicaid recipients	21.0
Public health measures: county mean ± SE (*n*)	
Alcohol abuse per 10,000 ED visits	47.7 ± 1.9
Substance abuse per 10,000 ED visits	63.6 ± 2.6
Suicide incidence per 100,000 population in 1 year	13.6 ± 0.4 (90)
% smoking in adults	24.0 ± 0.5
% hypertension in adults	29.0 ± 0.5 (89)
% diabetes mellitus in adults	11.3 ± 0.1
Infant mortality rate^a^	7.2 ± 0.2 (81)

*n* is specified when it differs from the number of counties in Indiana, 92

^a^Deaths in children less than 1 year of age per 1000 live births

**Table 2:** Indiana regional psychiatric workforce

	Total regional psychiatry	Geriatric psychiatry	Child-adolescent psychiatry	Addiction psychiatry	
Total counties	43	11	26	11	
Total psychiatrists	365	13	82	15	
Population to provider ratio	23,029	83,348	26,139	–	
Total FTE	282.9	10.4	61.1	11.8	
FTE per psychiatrist (mean ± SE)	0.78 ± 0.01	0.80 ± 0.09	0.75 ± 0.03	0.79 ± 0.08	*p* = 0.68

**Table 3:** Population characteristics for counties with vs without regional psychiatry FTE

	Counties with reported regional psychiatry FTE	Counties without reported regional psychiatry FTE
Number of counties	43	49
% of state population	81.5	18.5
County mean ± SE		
% population within rural area	36.1 ± 3.2	70.5 ± 3.0**
% population income less than 200% Federal Poverty Line	34.3 ± 1.0	35.2 ± 1.0
% Population age 65 or older	14.0 ± 0.3	15.8 ± 0.2**
% Population under age 18	24.4 ± 0.4	23.7 ± 0.3
% Medicaid recipients	19.3 ± 0.7	19.8 ± 0.6
Alcohol abuse	53.0 ± 2.7	43.1 ± 2.6*
Substance abuse	63.0 ± 3.9	64.1 ± 3.6
Suicide incidence	12.7 ± 0.6	14.4 ± 0.6 (*n* = 47)*
% smoking in adults	22.9 ± 0.7	25.0 ± 0.7*
% hypertension in adults	28.1 ± 0.7	29.8 ± 0.7 (*n* = 46)
% diabetes mellitus in adults	10.8 ± 0.2	11.9 ± 0.2*
Infant mortality rate^a^	7.0 ± 0.3	7.5 ± 0.3 (*n* = 38)

*n* is specified when it differs from the number of counties in a given category

Suicide incidence (per 100,000 population per 1 year)

Substance and alcohol abuse (per 10,000 ED visits)

*p≤0.05; **p<0.0001

^a^Deaths in children less than 1 year of age per 1000 live births

**Table 4:** Population characteristics by rurality

	Rural category 1	Rural category 2	Rural category 3	Rural category 4	*r*
% rural population range	0–25	> 25–50	> 50–75	> 75–100	
Total counties	18	20	33	21	–
% of state population	62.8	16.2	14.7	6.0	–
% population income less than 200% FPL	35.5	33.4	34.4	36.3	0.08
% population age 65 or older	57.8	18.3	16.9	6.9	0.53**
% population under age 18	63.1	16.2	14.6	6.0	− 0.03
% population within rural area	10.7	39.1	60.5	89.1	–
% population Medicaid recipients	22	19.6	18.9	19.1	− 0.06
Public health measures (county mean ± SE)					
Alcohol abuse	62.6 ± 4.7^a^	50.9 ± 3.9	45.3 ± 2.7	35.8 ± 2.8^a^	− 0.52**
Substance abuse	61.8 ± 3.5	69.2 ± 5.4	66.6 ± 5.2	55.0 ± 5.5	− 0.18
Suicide incidence	12.5 ± 0.7	12.9 ± 0.7	13.3 ± 0.7	15.8 ± 1.0 (*n* = 19)	0.25*
% smoking in adults	22.1 ± 1.0	23.3 ± 1.1	24.0 ± 0.6	26.4 ± 1.5	0.29*
% hypertension in adults	29.8 ± 0.9	28.1 ± 1.3	29.5 ± 0.7	28.2 ± 1.2 (*n* = 18)	− 0.06
% diabetes mellitus in adults	10.4 ± 0.4^a^	10.9 ± 0.3	11.9 ± 0.2^a^	11.7 ± 0.2	0.36*
Infant mortality rate^b^	6.4 ± 0.4	7.5 ± 0.3	7.4 ± 0.4 (*n* = 31)	7.6 ± 0.7 (*n* = 12)	0.19

* Denotes significant correlation using county level data stratified by % population within rural area, *p* ≤ 0.05

** Denotes significant correlation using county level data stratified by % population within rural area, *p* < 0.0001

Suicide incidence (per 100,000 population per 1 year)

Substance and alcohol abuse (per 10,000 ED visits)

*n* is specified when it differs from the number of counties in a given category

^a^Denotes significance in comparison to statewide county average, *p*≤0.05

^b^Deaths in children less than 1 yr. of age per 1000 live births

**Fig. 3:**
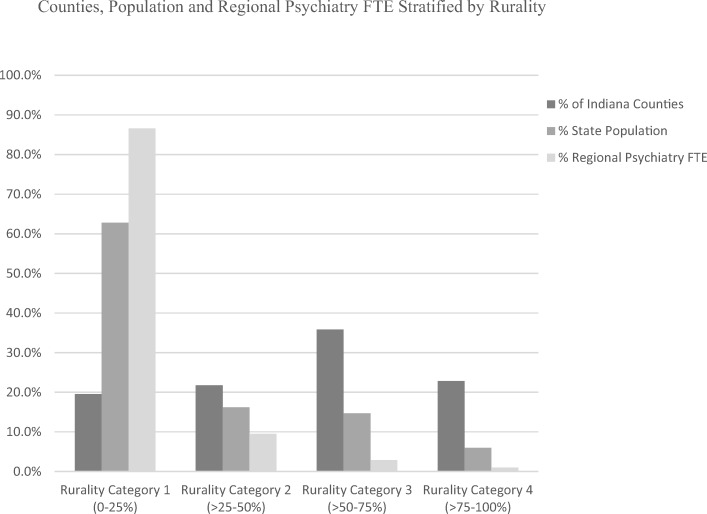
Counties, population, and regional psychiatry FTE stratified by rurality

Further evaluation stratified by proportion of rurality demonstrated that more than 85% of the reported regional psychiatry FTE was within the first rural category (0–25% rural population), and more than 95% was within the first two rural categories (0–50% rural population; Fig.3). A similar pattern of distribution was demonstrated regarding subspecialty practice in geriatric, child-adolescent, and addiction psychiatry (Table 5). Rurality categories 3 and 4 (50–75% rural population and 75–100% rural population) had population/psychiatry-FTE ratios more than seven times higher than rural category 1, and more than five times higher than the state-wide ratio (Tables 2 and 5).

**Table 5:** Psychiatric practice by rurality

	Rural category 1	Rural category 2	Rural category 3	Rural category 4
% rural population range	0–25	> 25–50	> 50–75	> 75–100
% total psychiatry FTE	86.6	9.5	2.9	1.0
Counties reporting practice	18/18	13/20	9/33	3/21
Population to psychiatry FTE	16,694	39,972	118,384	134,709
% of total geriatric psychiatry FTE	65.4	34.6	–	–
Counties reporting practice	8/18	3/20	0/33	0/21
Geriatric population/geriatric psychiatry FTE	73,699	44,197	–	–
% of total child-adolescent psychiatry FTE	87.9	8.5	3.6	–
Counties reporting practice	18/18	6/20	2/33	0/21
Youth population/child-adolescent psychiatry FTE	18,775	49,759	96,937	–
% of total addiction psychiatry FTE	88.1	11.9	–	–
Counties reporting practice	9/18	2/20	0/33	0/21

**Fig. 4:**
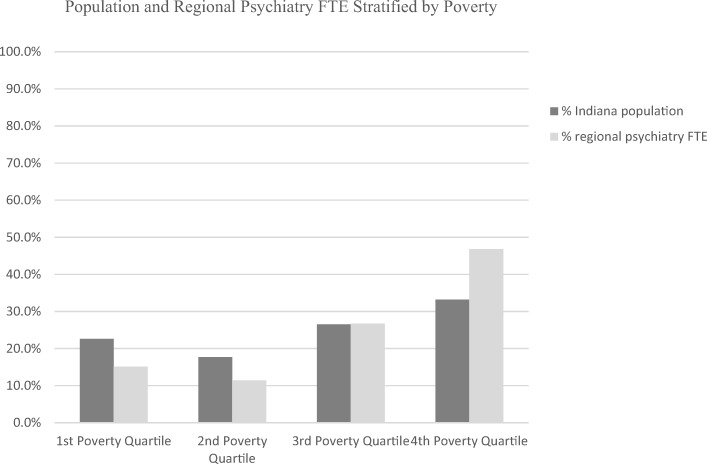
Population and regional psychiatry FTE stratified by poverty

In contrast to the data stratified by rurality, stratification by poverty (percent of population at or below 200% FPL) demonstrates a greater percentage of psychiatry FTE, and a lower population to psychiatry FTE ratio, in the third and fourth poverty quartiles (Table 6, Fig. 4, Supplemental Figure 2). The absolute differences in these measures were much smaller than for rurality. Subspecialty practice in geriatric, child-adolescent, and addiction psychiatry was more evenly distributed throughout the poverty quartiles than for rural categories. With the exception of suicide incidence, poverty had a positive correlation with all evaluated measures of public/behavioral health, indicating greater public/behavioral health burden in counties with a greater percent of people living at or below 200% FPL (Table 7). There was no correlation between poverty and rurality, though the first and second poverty quartiles had slightly higher percentages of rural populations. Details regarding the practice settings of regional psychiatrists stratified by both rurality and poverty quartile are presented in Supplemental Table 2.

**Table 6:** Psychiatric practice by poverty quartile

	1st poverty quartile	2nd poverty quartile	3rd poverty quartile	4th poverty quartile
% total psychiatry FTE	15.1	11.4	26.7	46.8
Counties reporting practice	12/23	6/23	14/23	11/23
Population/Psychiatry FTE	34,455	35,665	22,891	16,339
% of total geriatric psychiatry FTE	26.9	8.7	34.6	29.8
Counties reporting practice	3/23	2/23	4/23	3/23
Geriatric population/geriatric psychiatry FTE	66,706	178,333	70,461	85,740
% of total child-adolescent psychiatry FTE	21.6	6.2	26.4	45.8
Counties reporting practice	10/23	2/23	6/23	8/23
Youth population/child-adolescent psychiatry FTE	28,774	75,118	25,825	18,431
% of total addiction psychiatry FTE	16.9	16.9	34.7	31.4
Counties reporting practice	2/23	2/23	3/23	4/23

**Table 7:** Population characteristics by poverty quartile

	1st poverty quartile	2nd poverty quartile	3rd poverty quartile	4th poverty quartile	*r*
Number of counties	23	23	23	23	–
% of state population	22.6	17.7	26.5	33.2	–
% population income less than 200% FPL	23.0	33.7	37.2	42.1	–
% population age 65 or older	21.5	18.5	29.3	30.7	0.18
% population under age 18	23.8	17.9	26	32.3	N/A
% population within rural area	31.3	39.1	25.7	20.1	0.08
% population Medicaid recipients	13.5	20.4	22.5	25.2	0.72**
Public health measures (county mean ± SE)					
Alcohol abuse	38.9 ± 2.7b	44.2 ± 3.3	51.3 ± 3.9	56.5 ± 4.5	0.40*
Substance abuse	48.9 ± 2.7^c^	59.4 ± 4.7	75.4 ± 4.5^b^	70.5 ± 6.9	0.38*
Suicide incidence	12.4 ± 0.6	14.7 ± 1.0	13.9 ± 0.8	13.2 ± 0.9	0.14
% Smoking in adults	21.4 ± 1.0^b^	24.4 ± 1.1	25.5 ± 1.0	24.7 ± 1.0	0.34*
% hypertension in adults	28.3 ± 1.0 (*n* = 22)	28.2 ± 1.0 (*n* = 21)	29.1 ± 1.0	30.2 ± 1.0	0.24*
% diabetes mellitus in adults	10.6 ± 0.2^b^	11.7 ± 0.3	12.0 ± 0.3^b^	11.2 ± 0.3	0.29*
Infant mortality rate^a^	6.7 ± 0.4 (*n* = 20)	6.8 ± 0.4 (*n* = 17)	7.4 ± 3.6 (*n* = 22)	7.9 ± 0.5 (*n* = 22)	0.28*

* Denotes significant correlation using county level data stratified by % population within rural area, *p* < 0.05

** Denotes significant correlation using county level data stratified by % population within rural area, *p* < 0.0001

*n* is specified when it differs from the number of counties in a given category

N/A denotes a distribution that is not normal

Suicide incidence (per 100,000 population per 1 year)

Substance and alcohol abuse (per 10,000 ED visits)

^a^Deaths in children less than 1 year of age per 1000 live births

^b^Denotes significance in comparison to statewide county average, *p* < 0.05

^c^Denotes significance in comparison to statewide county average, *p* < 0.0001

The authors tested the utility of using suicide incidence and infant mortality rate as indicators of more general public/behavioral health in Indiana counties. Suicide incidence did have some correlation with other measures of behavioral health. For instance, there was a moderate correlation with substance abuse and smoking and a weaker correlation with alcohol abuse. However, suicide incidence had no correlation with infant mortality rate, diabetes prevalence, or hypertension prevalence (Table 8). Infant mortality rate had a weak correlation with diabetes, and no correlation with other measures of public/behavioral health. The evaluations stratified by poverty demonstrated that poverty had a more generalized correlation with various public/behavioral health measures (Table 7). Thus, the authors decided to further investigate the percent of Medicaid recipients as it is a closely related population characteristic. This evaluation demonstrates that Medicaid had a significant positive correlation with all other measures of public/behavioral health (Table 8).

**Table 8:** Correlation among measures of public/behavioral health

	Suicide incidence	Infant mortality rate^a^	% population Medicaid recipients
Alcohol abuse	0.23*	− 0.10	0.51**
Substance abuse	0.45**	0.09	0.59**
Smoking	0.47**	0.19	0.46**
Hypertension	0.13	− 0.09	0.33*
Diabetes mellitus	0.18	0.22*	0.43**
Infant mortality rate^a^	0.01	1	0.28*
Suicide incidence	1	0.01	0.39**

Hypertension, smoking, and diabetes mellitus measures are prevalence % adult population

Suicide incidence (per 100,000 population in 1 year)

*p ≤ 0.05; ** *p* < 0.0001

^a^Deaths in children less than 1 year of age per 1000 live births

## Discussion

Taken as a whole, these data indicate that rural populations of Indiana are grossly underserved regarding local psychiatric services. The magnitude of this disparity is alarming. While some rural residents with mental illness may commute to more urban/metropolitan areas for psychiatric services, the scarcity of colocation is an additional barrier to access. Those with mental illness already have many medical, social, and economic risks. Additional barriers to accessing care only compound the problem. The percent of people living at or below 200% poverty or insured under Medicaid does not differ greatly by rurality, thus rural populations likely face similar socioeconomic barriers (Table 4). These results align with findings from the National Comorbidity Survey Replication which indicate no rural-urban differences in the prevalence of the most mental illnesses (i.e., disorders such as PTSD, mood, anxiety, impulse control, and substance use disorders).^27^

While populations with greater rurality struggle with modestly higher rates of suicide, smoking, and diabetes, it is important to note that the magnitude of these differences are small in comparison to the five to seven times greater population to psychiatry FTE ratio. Furthermore, rural populations have much lower emergency department utilization for alcohol abuse. A similar pattern of distribution favoring urban/metropolitan areas likely exist for the physician workforce distribution as a whole, and further studies can address this question. This points to the noteworthy resiliency of rural populations. These results suggest rural populations offer much to learn from a public health and medical services efficiency perspective. While these data reflect a population that is resilient, it is important not to overlook their underrepresentation in terms of local psychiatric services. Increasing psychiatric services to rural populations should be approached carefully to not disrupt the interest and culture of these resilient people.

Alternately, it is important to consider the reasons why urban/metropolitan areas have a larger proportion of physiatrists. The majority of Indiana’s population resides in urban/metropolitan areas (Table 1). Therefore, proximity to a larger and less dispersed population of potential patients, greater opportunities to share call and consult with peer psychiatrists, as well as greater availability of other health care professionals for referrals and consults may contribute to distribution issues. In addition, personal factors may influence a psychiatrist decisions regarding practice location.^28^ Greater and more diverse employment and education opportunities for psychiatrists’ families in urban/rural communities may be another reason a great proportion of psychiatrists chose to practice in these communities. Overcoming or addressing these potential barriers is critical to addressing psychiatry workforce shortages in rural communities.

Counties with a greater percentage of people living at or below 200% poverty were identified as having higher rates of all public/behavioral health problems except for suicide incidence (Table 7). These Counties also had slightly better representation in terms of local psychiatric services (Fig. 4). This may point toward the relationship between poverty and health service utilization; however, further studies are needed to clarify the dynamics between health services utilization, poverty, and insurance status in Indiana.

This study demonstrated limited utility in using suicide incidence or infant mortality rate as indicators of a more general public/behavioral health status at a county level (Table 8). While suicide incidence did have significant positive correlation with measures of behavioral health, there was no correlation with other measures of public health. Surprisingly, infant mortality had no correlation with most measures of public/behavioral health. Of the factors evaluated in this study, percent of population on Medicaid was identified as the best indicator of adverse public/behavioral health outcomes. Taken together, these findings suggest that social factors have utility as indicators of public/behavioral health. Further studies are necessary to better understand this relationship.

The complexity of the mental health workforce in the USA is evident in the literature.^19,29^ Most patients with mental illness remain untreated, and the majority who receive treatment are getting it from providers other than psychiatrists.^30,31^ Several evidence-based strategies are demonstrated to improve the distribution and delivery of psychiatric services. These strategies will likely be most effective at addressing the complex issue of psychiatry workforce shortages as coordinated efforts rather than standalone initiatives.

Regarding distribution, monetary incentives are helpful in the recruitment of licensed psychiatrists, and loan repayment programs have increasing importance given unprecedented increases in medical school tuition.^32–34^ Selectivity in the allotment of monetary incentives regarding common background characteristics of applicants and the target community can have a marked effect on retention.^35^ Developments in telepsychiatry hold some utility in extending the reach of psychiatrists and facilitating collaborative care.^36,37^ However, substituting in-person psychiatrist-patient interaction with telecommunications is not without concern regarding community integration, mentoring, and interpersonal exchanges of affect.

Delivery system solutions can also extend psychiatry services across the population, although an integrated approach is important to maximize the delivery of services, retain providers, and ensure the continuity of improvements. A strategy with recent heightened interest is increased utilization of other mental health providers such as Advanced Practice Nurses and Physician Assistants.^17–19,21,29^ Maximizing the utility of other professionals is resourceful, and integration of psychiatrists with other professionals is customary given the complex medical and social characteristics of many with mental illness.^38^ Models of collaboration have gained favor within psychiatry, have been tested in randomized control studies, and have proven effective in some settings.^39,40^ Prior studies stemming from the IMPACT trial have demonstrated that the integration of psychiatrists into a primary care environment in a collaborative care model can reduce psychiatric symptoms,^39^ improve patient satisfaction,^39^ reduce medical expenditure,^40^ and gain a high level of physician satisfaction.^41^

Selective recruitment and training is an invaluable component to improving access to in-person services and ensuring the continuation of services to targeted populations.^42^ The basis is exposure, inclusion, and support for those most likely to serve a population of interest. Some empirical mechanisms to accomplish this goal in rural areas include (1) matriculating medical students with demographic backgrounds comparable to the targeted populations as well as expressed interest in serving those populations,^42,43^ (2) curriculum tracks in medical school with focus on rural health,^42^ (3) rural residency programs,^44^ and (4) the development of a rural medical school campus.^45^ In itself, addressing regional and sociodemographic disparities in the provision of psychiatric healthcare can play a critical role in recruitment and sustainability by exposing members of an underserved community to the practice of psychiatry. Integrating with a population of interest opens opportunities for mentoring and employment. Key components of collaborative care are education and participation in care^9^; thus, a collaborative model in itself may be a cost-effective mechanism to enhance recruitment and training where it is lacking. Finally, in order to substantially increase the overall psychiatry workforce, and facilitate transitions for potential candidates, it is essential to expand psychiatry residency training positions and the medical education infrastructure.

### Limitations

While this study strived to generate accurate, comprehensive, and cohesive data, there are recognized limitations in this study. The initial psychiatry workforce data were collected via voluntary survey at the time of physician license renewal. Primary data collection was performed to (1) identify psychiatrists who were among the non-respondents and (2) identify and/or verify primary practice location and average hours of clinical service.^46^ Although there were attempts to collect data from the entire psychiatry workforce population, this study includes only 91% of all licensed psychiatrists in Indiana. There were a number of psychiatrists for whom the authors were unable to verify information or had to be excluded for reasons presented in the methods section. Missing data is one recognized limitation. Additionally, psychiatrists practicing in the VA system are federal employees and are not required to hold a medical license within the State of Indiana. Therefore, the authors were unable to include psychiatrists practicing within the VA system in Indiana that did not hold an Indiana medical license.

Due to inherent limitations in verifying all secondary practice location characteristics, this study was restricted to the primary practice characteristics of respondents in order to improve accuracy. Of the psychiatrists able to be verified, 56 psychiatrists reported a total of 16.4 FTEs in secondary practices, which amounts to 5.8% of the primary practice FTEs. The verified secondary practice characteristics were very similar to the primary practice characteristics. The proportions of FTEs for subspecialty practice were also very similar to that of primary practice, with 21.6% being child-adolescent psychiatry, 4.2% being addiction psychiatry, but with no reported secondary practice in geriatric psychiatry. Eighty-four percent of the reported secondary practice FTE were within the first and second rural categories, and 75.6% were within the third and fourth poverty quartiles. Thus, while the overall quantity of regional psychiatry FTE is slightly greater than what was analyzed for this study, the patterns of distribution are not substantially altered.

Another barrier to this analysis is that the effect of regional population movement on public health outcomes or psychiatric services data could not be determined. As with the current study, it has been well documented that the majority of psychiatric services are located in urban populous areas. Thus, boundary regions in close proximity to major cities are likely to be more effected by this variable. The census classification for rural and urban areas is based on population density and not commuting patterns. The Office of Budget and Management (OBM) uses a classification of rurality influenced by commuting patterns, but this alternative approach has other limitations. For example, if 100% of the population within a county fit the census definition of rural but 25% of the employment in the county consists of workers commuting from a central metropolitan county, the county would be classified as urban. The authors chose to use a census-based classification system for rural vs urban because it provides more regional classification within the county than does the OBM classification system. This allowed for better characterization of regional practice characteristics of psychiatrists and further characterization of each county based on percentage of population residing within a rural area.

Data regarding the incidence of suicide in Indiana were extracted from a report released by the Indiana State Department of Health and is presented in this paper as a per-year average of data collected from 2006 through 2010. In two counties (Warren and Ohio), the data were suppressed for confidentiality because there were less than five recorded suicides during the period of data collection, thus these counties were excluded from further analysis. The time frame of data collection for other measures of public health ranged from 2006 through 2015. While the time period does not perfectly align with that of the psychiatry workforce data, the integration of such data is informative and largely novel.

Finally, the analyses of other public health data are limited to the availability, accuracy, and precision of the data collection. First, public health data on the prevalence of mental health conditions are limited and were not available for this study. Therefore, the authors are unable to make any conclusions regarding the association between psychiatry workforce capacity and population mental health conditions. Additionally, the study team was not directly involved in the collection of the data that were included in the study, but the data were obtained from reputable resources with recognized expertise. Future studies may benefit from enhanced collection of data on mental health conditions and improved techniques of public data collection and refinement.

## Implications for Behavioral Health

These findings have important implications for researchers, educators, and policy makers. First, health services research is dependent upon the availability of high-quality data to support analyses. The behavioral health workforce is critical to the delivery of behavioral health services within organizations and across populations. This workforce is comprised of a number of licensed occupations for which licensing agencies/entities maintain data. In the USA, a number of states, including Indiana, have enacted policies to enhance health workforce data for the purpose of informing workforce policy, evaluation, and research.^47,48^ Behavioral health services researchers in the USA and internationally should explore the development of strategic relationships and partnerships with professional licensing agencies/entities in order to leverage workforce data for research that informs policy and advances population health. Additionally, educators along the psychiatry workforce pipeline from primary education to graduate medical education need to be aware of and align with the needs of the communities they serve. Primary and secondary educators in rural communities can enhance the pipeline by encouraging students to explore careers in psychiatry and behavioral health. Medical schools located in states with rural workforce shortages should consider strategies to enhance admissions of high-quality candidates with a rural background. Any expansion efforts for Graduate Medical Education must consider and prioritize the need for additional psychiatrists, especially in rural communities. Finally, policy makers have an important role in developing the workforce and education policy. Addressing the shortage of psychiatrists in rural communities around the globe will not be solved at the state, national, or international level. It will require local advocates and coordinated local solutions. Policy makers should strive to become familiar with the behavioral health needs of their constituency and workforce needs within their respective districts. They should seek to develop and advocate for policy solutions that align with the unique needs of their communities. Pervasive and persistent behavioral workforce shortages plague communities across the country. By “digging deeper” and leveraging state level workforce and public health data at a state level, this study identified that although rural communities experience much greater shortages of psychiatrists, their mental health associated outcomes are comparable to their urban/metropolitan counterparts. These findings suggest a resiliency in rural communities. As states implement strategies to increase psychiatric services within rural communities, care must be taken to not disrupt the interest and culture of these resilient people.

## Electronic supplementary material

ESM 1 (DOCX 476 kb)
