# Supplementary material for: Scratching the Surface of Psychiatric Services Distribution and Public Health: an Indiana Assessment
Source: J Behav Health Serv Res. 2018 Jun 26;46(2):267–82. doi: 10.1007/s11414-018-9626-7 (PMC6470314; doi:10.1007/s11414-018-9626-7)

Supplemental Materials

| **Supplemental Table 1**  Measurements and Definitions | | |
| --- | --- | --- |
|  | **Variable** | **Definition** |
| **Population Characteristics** | Total population | Count of total civilians residing in a specified region (i.e., state, county) |
|  | % Population residing in rural area | Proportion of the total population in a specified region who reside in a rural area |
|  | Rurality Quartile | Quartile categories for percent of population residing in a rural area:  1=less than 25% of population reside in a rural area  2=25% – 50% of population reside in a rural area  3=50% - 75% of population reside in a rural area  4=more than 75% of population reside in a rural area |
|  | % Income less than 200% Federal Poverty Line | Proportion of the total population in a specified region whose income is less than 200% the Federal Poverty Line (FPL) |
|  | Poverty Quartile | Quartile categories for the proportion of the population whose incomes in less than 200% FPL  1=less than 25% of population’s incomes is below 200% FPL  2=25% – 50% of population’s incomes is below 200% FPL  3=50% - 75% of population’s incomes is below 200% FPL  4=more than 75% of population’s incomes is below 200% FPL |
|  | % Population age 65 or older | Proportion of the total population in a specified region who is aged 65 years or older |
|  | % Population under 18 yrs. of age | Proportion of the total population in a specified region who is less than 18 years old. |
|  | % Medicaid recipients | Proportion of the total population in a specified region who are Medicaid |
| **Public Health Measures** | Alcohol Abuse | Rate measuring the number of the ED discharges associated with alcohol abuse per 10,000 ED visits |
|  | Substance Abuse | Rate measuring the number of the ED discharges associated with substance abuse per 10,000 ED visits |
|  | Suicide Incidence | Number of suicide deaths per 100,000 persons per year |
|  | % Smoking in adults | Proportion of the adults who reported smoking every day or most days and has smoked at least 100 cigarettes in their lifetime |
|  | % Hypertension in adults | Proportion of the population who reported having high blood pressure |
|  | % Diabetes Mellitus in adults | Proportion of the adult population who report being diagnosed with diabetes mellitus |
|  | Infant Mortality Rate | Number of infant deaths (children less than 1 year old) per 1,000 live births. |
| **Psychiatrist Workforce Characteristics** | Total FTE | Sum of FTE for all psychiatrists actively practicing in a specified region |
|  | FTE per psychiatrist | Average FTE for a psychiatrist, calculated by dividing the total FTE by the total number of psychiatrists actively practicing in a specified region. |
|  | Population per provider ratio | Total population in a specified region divided by the total FTE for all psychiatrists actively practicing in the same region |
|  | % total psychiatry FTE | Proportion of total psychiatrists actively practicing in a specified region |
|  | % of total geriatric psychiatry FTE | Proportion of psychiatrist with a specialty in geriatric psychiatrist actively practicing in a specified region |
|  | % of total child-adolescent psychiatry FTE | Proportion of psychiatrists with a specialty in child and adolescent psychiatry actively practicing in a specified region |
|  | % of total addiction psychiatry FTE | Proportion of psychiatrists with a specialty in addiction psychiatry actively practicing in a specified region |

| **Supplemental Table 2** | | | | | |
| --- | --- | --- | --- | --- | --- |
| Summary of Regional Psychiatrists Practice Setting: FTE (% Categorical FTE) | | | | | |
| **Rural Category** | 1 | 2 | 3 | 4 | Statewide Sum |
| Office/Clinic - Private Practice | 102.5 (47.2%) | 10.7 (43.0%) | 3.2 (45.1%) | 0 | 116.4 (46.2%) |
| Hospital | 70.8 (32.6%) | 7.6 (30.5%) | 3 (42.3%) | 1 (34.5%) | 82.4 (32.7%) |
| Other Specified | 29.7 (13.7%) | 4.1 (16.5%) | 0.9 (12.7%) | 0 | 34.7 (13.8%) |
| Unspecified | 9.0 (4.1%) | 2.5 (10.0%) | 0 | 1.9 (65.5%) | 13.4 (5.3%) |
| Medical School | 5.3 (2.4%) | 0 | 0 | 0 | 5.3 (2.1%) |
| Categorical Sum | 217.3 | 24.9 | 7.1 | 2.9 | 252.2 (100%) |
| Non-Respondents | 27.7 | 2 | 1 | 0 | 30.7 |
| Total FTE | 245.0 | 26.9 | 8.1 | 2.9 | 282.9 |
|  |  |  |  |  |  |
| **Poverty Quartile** | 1 | 2 | 3 | 4 | Statewide Sum |
| Office/Clinic - Private Practice | 24.6 (68.0%) | 12.2 (40.0%) | 27.3 (41.3%) | 52.3 (43.8%) | 116.4 (46.2%) |
| Hospital | 5.0 (13.8%) | 9.8 (32.1%) | 27.8 (42.1%) | 39.8 (33.3%) | 82.4 (32.7%) |
| Other Specified | 4.9 (13.5%) | 7.6 (24.9%) | 7.7 (11.6%) | 14.5 (12.1%) | 34.7 (13.8%) |
| Unspecified | 1.7 (4.7%) | 0.9 (3.0%) | 3.3 (5.0%) | 7.5 (6.3%) | 13.4 (5.3%) |
| Medical School | 0 | 0 | 0 | 5.3 (4.4%) | 5.3 (2.1%) |
| Categorical Sum | 36.2 | 30.5 | 66.1 | 119.4 | 252.2 (100%) |
| Non-Respondents | 6.5 | 1.8 | 9.4 | 13 | 30.7 |
| Total FTE | 42.7 | 32.3 | 75.5 | 132.4 | 282.9 |
| –Office/Clinic - Private Practice: corresponding to survey selection Office/Clinic-(Solo practice, Partnership, Single Specialty Group, and Multiple Specialty Group) | | | | | |
| –Hospital: corresponding to survey selection Hospital (Inpatient, Outpatient and Ambulatory Care Center) | | | |  |  |
| –Other Specified: corresponding to survey selection Federal Government Hospital, Federal/State/Community Health Center, Telemedicine, Nursing Home or Extended Care Facility, Home Health Setting, Hospital-Emergency Department | | | | | |
| –Unspecified: practice setting not determined | |  |  |  |  |
| –Medical School: corresponding to survey selection Medical School | | |  |  |  |
|  |  |  |  |  |  |


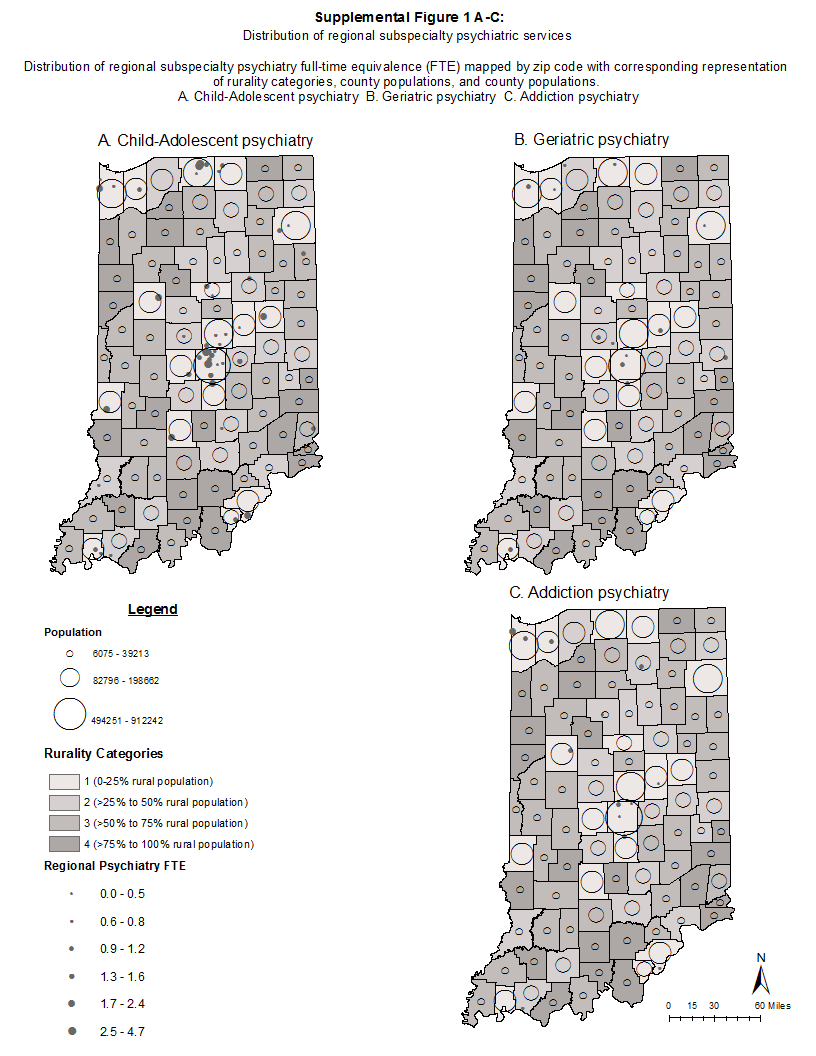


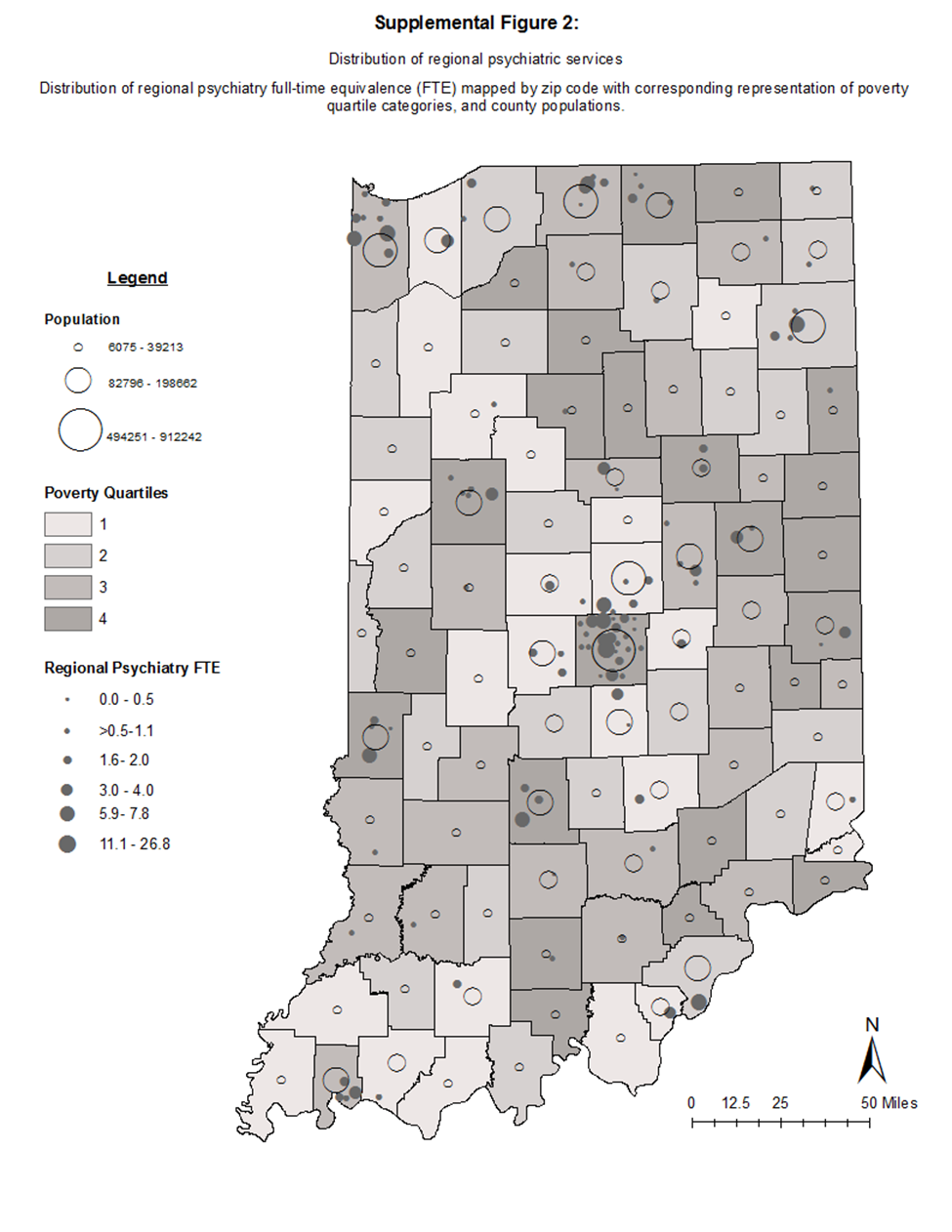

Supplement: Supplementary file 1 — (DOCX 476 kb) [file 11414_2018_9626_MOESM1_ESM.docx]
